# Key pathways and genes that are altered during treatment with hyperbaric oxygen in patients with sepsis due to necrotizing soft tissue infection (HBOmic study)

**DOI:** 10.1186/s40001-023-01466-z

**Published:** 2023-11-10

**Authors:** Julie Vinkel, Leonor Rib, Alfonso Buil, Morten Hedetoft, Ole Hyldegaard

**Affiliations:** 1 Department of Anesthesiology, Copenhagen University Hospital, Rigshospitalet, Inge Lehmanns Vej 6, 2100 Copenhagen, Denmark; 2 Department of Clinical Medicine, University of Copenhagen, Copenhagen, Denmark; 3 Biotech Research and Innovation Centre, University of Copenhagen, Copenhagen, Denmark; 4 Institute for Biological Psychiatry, Mental Health Centre Sct. Hans, Roskilde, Denmark; 5 Department of Anesthesiology, Zealand University Hospital, Køge, Denmark

**Keywords:** Necrotizing soft tissue infection, Hyperbaric oxygen treatment, Sepsis, Transcriptomics, Systems medicine, Differential gene expression, Inflammation, Immune cells

## Abstract

**Background:**

For decades, the basic treatment strategies of necrotizing soft tissue infections (NSTI) have remained unchanged, primarily relying on aggressive surgical removal of infected tissue, broad-spectrum antibiotics, and supportive intensive care. One treatment strategy that has been proposed as an adjunctive measure to improve patient outcomes is hyperbaric oxygen (HBO_2_) treatment. HBO_2_ treatment has been linked to several immune modulatory effects; however, investigating these effects is complicated due to the disease's acute life-threatening nature, metabolic and cell homeostasis dependent variability in treatment effects, and heterogeneity with respect to both patient characteristics and involved pathogens. To embrace this complexity, we aimed to explore the underlying biological mechanisms of HBO_2_ treatment in patients with NSTI on the gene expression level.

**Methods:**

We conducted an observational cohort study on prospective collected data, including 85 patients admitted to the intensive care unit (ICU) for NSTI. All patients were treated with one or two HBO_2_ treatments and had one blood sample taken before and after the intervention. Total RNAs from blood samples were extracted and mRNA purified with rRNA depletion, followed by whole-transcriptome RNA sequencing with a targeted sequencing depth of 20 million reads. A model for differentially expressed genes (DEGs) was fitted, and the functional aspects of the obtained set of genes was predicted with GO (Gene Ontology) and KEGG (Kyoto Encyclopedia of genes and Genomes) enrichment analyses. All analyses were corrected for multiple testing with FDR.

**Results:**

After sequential steps of quality control, a final of 160 biological replicates were included in the present study. We found 394 protein coding genes that were significantly DEGs between the two conditions with FDR < 0.01, of which 205 were upregulated and 189 were downregulated. The enrichment analysis of these DEGs revealed 20 GO terms in biological processes and 12 KEGG pathways that were significantly overrepresented in the upregulated DEGs, of which the term; “adaptive immune response” (GO:0002250) (FDR = 9.88E-13) and “T cell receptor signaling pathway” (hsa04660) (FDR = 1.20E-07) were the most significant. Among the downregulated DEGs two biological processes were significantly enriched, of which the GO term “apoptotic process” (GO:0006915) was the most significant (FDR = 0.001), followed by “Positive regulation of T helper 1 cell cytokine production” (GO:2000556), and “NF-kappa B signaling pathway” (hsa04064) was the only KEGG pathway that was significantly overrepresented (FDR = 0.001).

**Conclusions:**

When one or two sessions of HBO_2_ treatment were administered to patients with a dysregulated immune response and systemic inflammation due to NSTI, the important genes that were regulated during the intervention were involved in activation of T helper cells and downregulation of the disease-induced highly inflammatory pathway NF-κB, which was associated with a decrease in the mRNA level of pro-inflammatory factors.

*Trial registration:* Biological material was collected during the INFECT study, registered at ClinicalTrials.gov (NCT01790698).

**Supplementary Information:**

The online version contains supplementary material available at 10.1186/s40001-023-01466-z.

## Background

Necrotizing soft tissue infections (NSTI) are severe infections of the soft tissue surrounding the bones, and these infections appears to be rising on a global scale [1, 2]. Severe scarring, amputations, sepsis and its accompanying shock, and multi-organ failure are all linked to NSTIs [1, 3]. These systemic manifestations of severe infections are today understood as maladaptive changes in circulatory, cellular, and metabolic functions resulting in a dysregulated host response [4, 5]. Adjunctive immune modulating therapies, such as immunoglobulins, are used and have been tested for group A streptococcus NSTI infections. In addition, hyperbaric oxygen treatment (HBO_2_) is one treatment strategy that has been proposed as an adjunctive measure to improve patient outcomes in severe infections, particularly in NSTIs, with resultant improved survival and lower amputation requirements [6, 7]. HBO_2_ treatment entails intermittent breathing of 100% oxygen while under increased atmospheric pressure. Because the resulting high oxygen partial pressure is in blood plasma solution, it can reach physically obstructed areas where red blood cells are unable to pass allowing tissue oxygenation even when hemoglobin oxygen carriage is impaired [8]. When the NSTI diagnosis was first introduced, these infections were thought to be caused primarily by bacteria that thrived in anaerobic conditions, and HBO_2_ treatment was used to increase oxygen supply to anaerobes, with the goal of halting the infection [9, 10]. However, later research reveals that the effects of HBO_2_ treatment go far beyond simply changing the oxygen environment for the growing invading organisms, and that HBO_2_ treatment has been linked to a variety of immune modulatory effects, presumably due to its ability to promote changes in the oxidation–reduction (redox) balance in cells [11]. A systematic review of 58 studies on human tissue indicated that HBO_2_ treatment inhibited the pro-inflammatory transcription factor nuclear factor kappa B (NF-κB), decreased secretion of the cytokines IL-1, IL-6, and IL-8, and promoted an anti-inflammatory state overall [12]. This may explain why HBO_2_ treatment has been linked to improve survival in individuals with sepsis brought on by NSTIs [13–16]. However, key cytokines like IL-10 and TGF-β have been linked to both a protective effect and no effect of HBO_2_ treatment in sepsis [12, 17], and the effect of important transcription factors such as hypoxia inducible factors and NF-κB appears to be dependent on both the preconditional stage of hypoxia and inflammation, as well as the timing and number of consecutive hyperbaric sessions [11, 18]. This heterogeneous complexity may be the reason why efforts to identify disease-progression markers to track the effectiveness of treatment have been futile [19]. The emergence of new technical platforms makes it possible to characterize collections of biological molecules, which encourages researchers to cast a wider net when drawing conclusions regarding illnesses and cures. We conducted a functional enrichment analysis of all genes that were differentially expressed in whole blood samples before and after HBO_2_ treatment to better understand the underlying biological processes of HBO_2_ treatment effects in patients with NSTI.

## Methods

### Experimental approach

The current paper presents the findings of the observational HBOmic study based on prospectively collected data. A brief description of methods is provided in this paper, additional method description is available in the HBOmic study protocol [20] and in Additional file 1: Methods. Eighty-five patients submitted to ICU for NSTI were enrolled in the present study. All patients have been treated with HBO_2_ treatment and had one blood sample taken before and after the intervention. The HBO_2_ treatment consisted of breathing 100% oxygen at a pressure of 284 kPa without air-breaks with a compression period lasting 5 min and a decompression rate of 15 min. Patients received one or two sessions within the first 24 h of admission and the treatment duration of each HBO_2_ treatment session was 90 min.

### Data collection

**Fig. 1:**
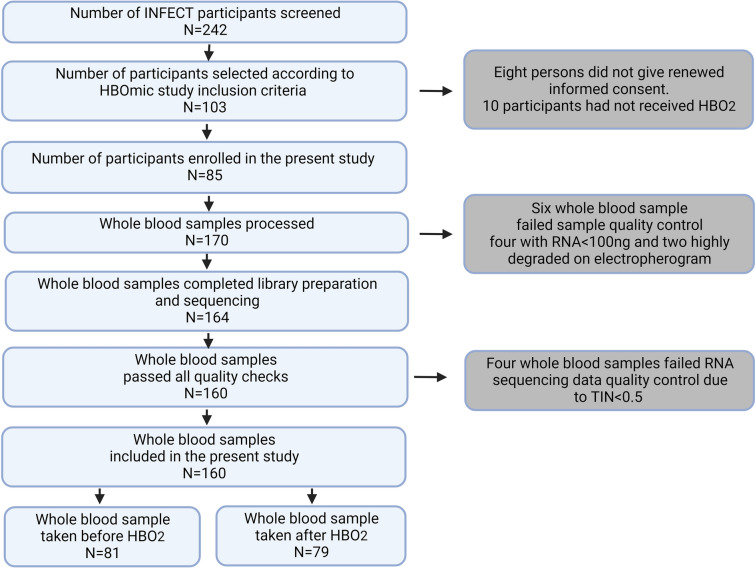
Study flow diagram. *INFECT* Systems Medicine to Study NSTI, *TIN* transcript integrity number, *HBO*_*2*_ hyperbaric oxygen treatment

Total RNAs from blood samples were extracted, and mRNA purified with rRNA depletion was performed as planned and described in the published study protocol [20]. Whole-Transcriptome RNA Sequencing was performed on the Illumina Novaseq6000 platform with a targeted sequencing read depth of 20 million reads. After sequential steps of quality control, a final of 160 whole blood samples from 83 patients were included in the present study (Fig. 1). More details on are provided in supplementary methods [see Additional file 1: Quality control].

### Sample size

The genome-wide transcriptional response to HBO_2_ treatment has not been addressed by other research groups. We performed a pilot study prior to the current study [20] and estimated the sample size needed to find a significant difference in gene expression in whole blood samples between before and after HBO_2_ treatment by published methods [43]. Assuming a coefficient of variation of 0.34, a depth of coverage of human reads of 218, a risk of type I error of 5%, and a risk of type II error of 20% (power 80%), the number of participants per group required is 38 to detect a 25% difference between conditions, giving a total sample size of 76 participants. We predicted that about 10% of samples would fail quality control and decided to include 85 participants in the study. As expected, in the current study, we focused on the genes that performed better, so we anticipate a higher power than that estimated from all transcripts in the pilot [40].

### Data preparation

The obtained reads were trimmed and aligned to the human genome using STAR [21]. These genes were normalized, and the data were fit to a gene-wise generalized linear model that included the following co-variates; patient identifier and a variable indicating whether a given samples was collected before or after HBO_2_ treatment (Additional file 1: Methods and Fig. S1). Differential gene usage was assessed by quasi-likelihood tests and adjusted for multiple comparisons with false discovery rate. The obtained data set of differentially expressed genes (DEGs) was separated in protein coding genes and other gene products using GENECODE release 43. Data preparation was performed with the edgeR package from Bioconductor in software R, version 4.0 [22]. The supplementary methods provide more details on the differential expression analysis [see Additional file 1: Data pre-processing and Differential expression].

### Functional enrichment analysis

For genes that were differential expressed between before and after HBO_2_ treatment, functional enrichment analysis was performed using “Gene Ontology” (GO) and “Kyoto Encyclopedia of Genes and Genomes” (KEGG) pathway enrichment analyses, to interpret gene functional annotation and functional enrichment.

### Gene Ontology (GO)

The GO knowledgebase is the world’s largest source of information on the function of genes [23]. The ontology is a set of terms with defined relationships (i.e., GO terms). When annotating the gene to its gene ontology the associations between gene products and the GO term is investigated. We used the GO annotation to determine which biological processes were implicated in our observable trait of DEGs between the two conditions: before and after treatment with HBO_2_.

### Kyoto Encyclopedia of Genes and Genomes (KEGG)

KEGG is an integrated database resource generated from 15 publicly available resources, mostly from National Center for Biotechnology Information RefSeq and GenBank and annotated by KEGG in the form of KEGG Orthology [24]. The collection is supplemented with a KEGG original collection of functionally characterized proteins from published literature. KEGG is a reference resource for interpreting high-level functions of the biological system from large-scale molecular datasets generated by high-throughput experimental technologies. KEGG analysis was aimed at exploring the possible key regulatory pathways for the enrichment of DEGs between the two conditions; before and after treatment with HBO_2_.

### Bioinformatic tools

We used the Database for Annotation, Visualization and Integrated Discovery (DAVID) 6.8 to perform GO and KEGG annotation of protein coding DEGs [25]. DAVID is a free, online bioinformatics resource that lists a comprehensive set of functional annotations that can be used to identify the biological significance of a list of genes. The enriched biological processes were analyzed with the DAVID default setting “BP DIRECT”. GO terms and KEGG pathways with a false discovery rate < 0.05, as calculated by the FDR adjustment method, were considered significant for the enrichment analysis. Further analyses are described in supplementary methods [see Additional file 1: Annotation of differentially expressed genes]. All protein-coding genes that were differentially expressed was used as background. For figures on enrichments results, we used the GOplot 1.0.2 R package [26] and the ShinyGO version 0.77 bioinformatic toll [27].

### Up- and down-regulated single markers

To further explore the effects of HBO_2_ treatment we screened all protein-coding DEGs between the two conditions for pro- and anti-inflammatory factors as well as their receptors and antagonists at a significance level of FDR > 0.05.

## Results

### Patient and sample characteristics

The patient characteristics are presented in Table 1. A detailed description of the patient population has been published previously [3]. The samples collected before HBO_2_ treatment were collected after a median of 32 h and 45 min (16 h and 1 min–79 h and 12 min) from first hospital admission with the NSTI diagnosis, a median of 4 h and 24 min (3 h–7 h 30 min) from arrival at a specialized hospital, and a median of 7 min (2–13 min) before the intervention with HBO_2_. The follow-up samples were collected after a median of 5 min (1–10 min) from the intervention with HBO_2_.

**Table 1:** Patient characteristics

Characteristics, N = 83	
Age	61 (52–69)
Sex, female	28 (34)
BMI	26 (23–31)
Comorbidities	
Cardiovascular disease	43 (52)
Diabetes	29 (35)
Peripheral vascular disease	10 (12)
COPD	8 (9.6)
Chronic kidney disease	6 (7.2)
Malignancy	5 (6)
Immune deficiency	3 (3.6)
Rheumatoid disease	3 (3.6)
Liver cirrhosis	2 (2.4)
Disease severity measures	
Septic shock on admission	34 (41)
SAPS II*	43 (35 −48)
SOFA, day 1†	8 (6–9)
LRINEC‡	8 (6–9)
Prognosis	
Days hospitalized in the intensive care unit	9 (5–14)
Amputation within seven days	10 (12)
Mortality, day 90§	11 (13)

Continuous data are presented as medians (IQR) and categorical data as absolute numbers (percentage)

*BMI* body mass index, *COPD* chronic obstructive pulmonary disease, septic shock on admission, defined as lactate > 2 mmol/L and use of vasopressor or inotrope, *LRINEC* Laboratory Risk Indicator for Necrotizing Fasciitis Score, *NSTI* necrotizing soft-tissue infection, *SAPS II* Simplified Acute Physiology Score II, *SOFA* Sequential Organ Failure Assessment

^*^Data were missing for two (2.4%) patients

^†^Data were missing for two (2.4%) patients

^‡^Data were missing for 11 (13.3%) patients

^§^Data were missing for one (1.2%) patient

In the present study, we found that the RNA and mRNA extracted by the above-described procedures were of the same high quality as found in the pilot study [20]. The various quality measures obtained are provided in the supplementary results [see Additional file 2,: Quality control].

### Differential expressed genes

We identified 394 protein coding genes that were significantly differentially expressed between the two conditions, before and after HBO_2_ treatment with FDR < 0.01. A histogram of the obtained *P*-values is shown in supplementary results [see Additional File 2: Fig. S2]. In comparison to before the intervention, 205 protein coding genes were upregulated and 189 were downregulated after HBO_2_ treatment, with a log2-fold change ranging between 0.712 and 0.668, as illustrated in the volcano plot in supplementary results [see Additional file 2: Fig. S3].

### Functional gene enrichment analysis

**Fig. 2:**
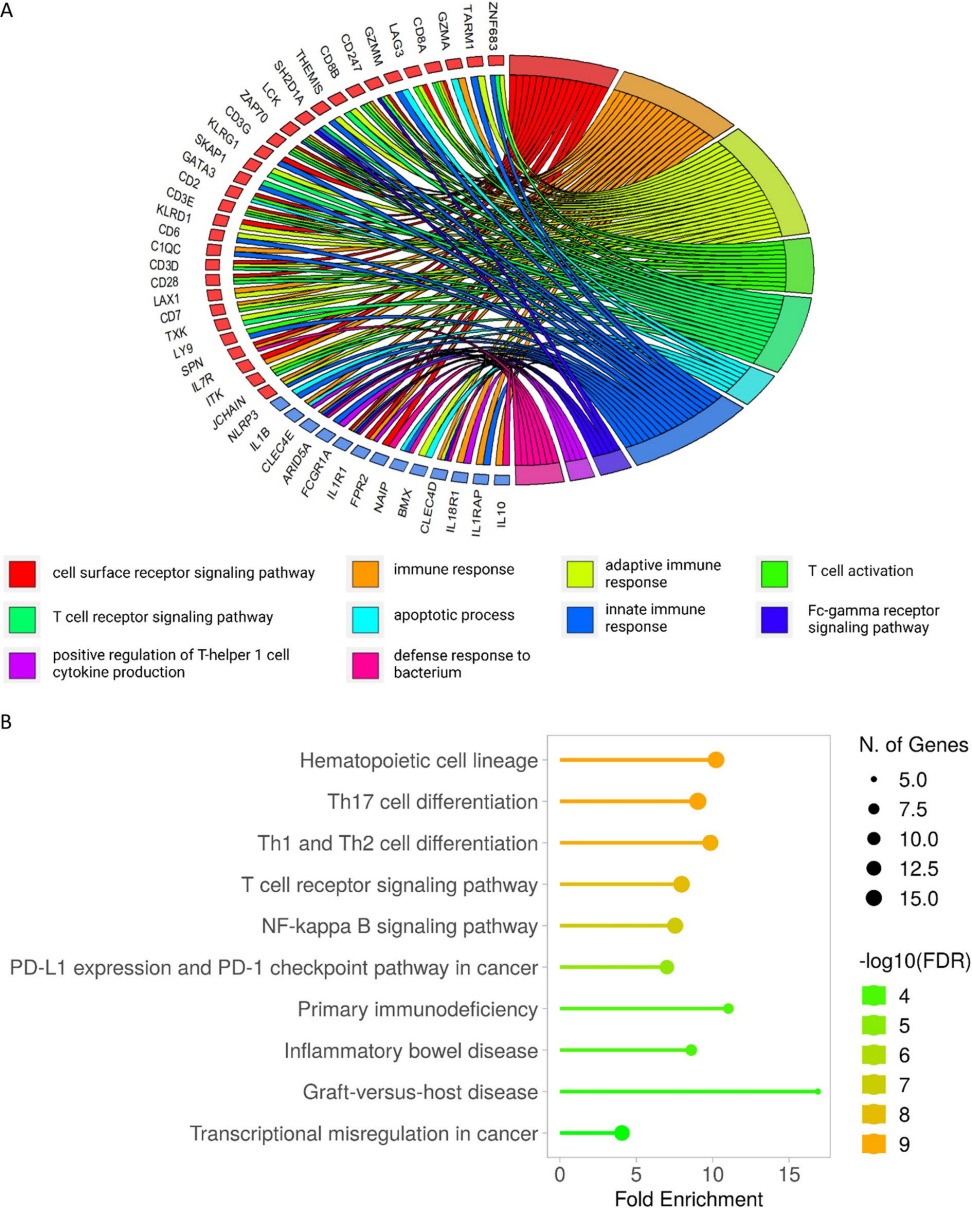
Enriched GO terms and KEGG pathways among differentially expressed genes. **A** GOchord plot of top 10 GO terms sorted by FDR. Only genes that are assigned to a minimum of 2 terms are displayed. Genes are ranked according to logFC, with red colors signifying upregulated genes and blue colors signifying downregulated genes. Genes symbols are used to describe genes. **B** Top 10 KEGG pathways sorted by FDR. Color code indicates -10log(FDR); the length of the bar indicates fold enrichment defined as the percentage of differentially expressed genes belonging to the pathway, divided by the corresponding percentage in the background. The size of the bubbles indicates the number of genes in the enriched pathway

The KEGG and GO enrichment analysis of all the genes that were differentially expressed between before and after HBO_2_ treatment with FDR < 0.01 found that the DEGs were primarily involved in a broad range of immunological processes concerning hematopoietic cell differentiation, proliferation, and apoptotic selection, as well as inflammatory conditions (Fig. 2).

The separate enrichment analysis of the DEGs that were significantly up- and down-regulated, revealed 20 GO terms in the category biological process that were significantly overrepresented in the upregulated DEGs, of which the term; “adaptive immune response” (GO:0002250) was the most significant (FDR = 9.88E-13). Other important biological processes associated with these upregulated DEGs were “T cell activation” (GO:0042110) and “T cell receptor signaling pathway” (GO:0050852) (Table 2). The KEGG enrichment analysis of the upregulated DEGs found 12 overrepresented pathways, with the “T cell receptor signaling pathway” (hsa04660) being the most significant (FDR = 1.20E-07), followed by “Th1 and Th2 cell differentiation” and “Th17 cell differentiation” (Table 2).

**Table 2:** Enriched biological processes and pathways from upregulated genes. Results from gene enrichment analysis with Kyoto Encyclopedia of Genes and Genomes (KEGG) and Gene Ontology (GO). All differentially expressed genes with FDR < 0.01 and positive fold change were included in the analysis. Percent is the percentage of differentially expressed genes belonging to the pathway. *P* value is the significance level of the enrichment. Genes are the gene symbol of the differentially expressed genes belonging to the pathway. FDR is the significance level after correction for multiple testing with false discovery rate﻿﻿

Category	Term	Percent	P value	Genes	FDR
Upregulated GO terms (top 3)					
BIOLOGICAL PROCESS	Adaptive immune response	11%	8.01E-16	EOMES, TRAT1, SH2D1A, TARM1, CAMK4, ZNF683, ZAP70, KLRD1, CD3D, CD3E, LAX1, CD3G, ITK, SKAP1, CD247, JCHAIN, THEMIS, CD6, CD7, CD8A, CD8B, TXK, LAG3	9.88E-13
BIOLOGICAL PROCESS	T cell activation	7%	1.45E-14	DPP4, CD28, NLRC3, RASGRP1, ZAP70, CD2, CD3E, CD3G, ITK, CD7, CD8A, LAG3, CD8B, LY9	8.98E-12
BIOLOGICAL PROCESS	T cell receptor signaling pathway	8%	4.45E-14	GATA3, CD28, ZNF683, ZAP70, BTN3A3, CD3D, CD3E, CD3G, ITK, PLCG1, CD247, SKAP1, THEMIS, LCK, CD8A, CD8B, TXK	1.83E-11
Upregulated KEGG pathways (top 3)					
PATHWAY	T cell receptor signaling pathway	7%	6.45E-10	NFATC2, CD28, RASGRP1, ZAP70, CARD11, CD3D, CD3E, CD3G, ITK, PLCG1, CD247, LCK, CD8A, CD8B	1.20E-07
PATHWAY	Th1 and Th2 cell differentiation	6%	1.44E-09	RUNX3, GATA3, NFATC2, IL2RB, ZAP70, CD3D, CD3E, CD3G, PLCG1, CD247, STAT4, TBX21, LCK	1.34E-07
PATHWAY	Th17 cell differentiation	6%	9.73E-09	GATA3, NFATC2, IL2RB, ZAP70, RORA, RORC, CD3D, CD3E, CD3G, PLCG1, CD247, TBX21, LCK	6.04E-07

GO enrichment analysis of the downregulated DEGs found two biological processes that were significantly enriched, of which the GO term “apoptotic process” (GO:0006915) was the most significant (FDR = 0.001), followed by “Positive regulation of T helper 1 cell cytokine production” (GO: 2000556). “NF-kappa B signaling pathway” (hsa04064) was the only KEGG pathway that was significantly overrepresented in the dataset of significantly downregulated DEGs (Table 3).

**Table 3:** Enriched biological processes and pathways from downregulated genes. Results from gene enrichment analysis with Kyoto Encyclopedia of Genes and Genomes (KEGG) and Gene Ontology (GO). All differentially expressed genes with a cut off FDR < 0.01 and negative fold change were included in the analysis. Percent is the percentage of differentially expressed genes belonging to the pathway. *P* Value is the significance level of the enrichment. Genes are the gene symbol of the differentially expressed genes belonging to the pathway. FDR is the significance level after correction for multiple testing with false discovery rate

Category	Term	Percent	P value	Genes	FDR
Downregulated GO terms (significant)					
Biological process	Apoptotic process	12%	7.82E + 08	IL1B, RNF144B, STK3, DRAM1, CASP5, MAP2K6, DDIT4, PLK3, GADD45Α, PLSCR1, MARCKS, HIP1, KLLN, BMX, BCL2A1, NLRP3, KIF1B, NFKBIA, TNFAIP3, TNFRSF10D, NR2E1, NAIP	0.001037
Biological process	Positive regulation of T helper 1 cell cytokine production	2%	5.62E + 10	ARID5A, IL1B, IL18R1, IL1R1	0.03726
Downregulated KEGG pathways (significant)					
PATHWAY	NF-kappa B signaling pathway	5%	7.17E-06	IL1B, TNFSF13B, IL1R1, BCL2A1, CXCL1, ICAM1, TNFAIP3, NFKBIA, TRIM25, GADD45Α	0.001147

Additional enriched biological GO terms and KEGG pathways can be found in supplementary results, illustrating all results from DEGs that are differentially expressed with FDR < 0.02 [see Additional file 2: Tables S1–S4].

### Differentially expressed pro- and anti-inflammatory markers

Aside from the markers in the above-mentioned enriched terms and pathways, single factors related to inflammatory processes were up- or downregulated from before to after HBO_2_ treatment, these are shown in supplementary results and included one cytokine, three chemokines, 18 ligand receptors, and one cell adhesion protein [see Additional file 2: Table S5].

## Discussion

The present study used gene enrichment analysis to predict the functions of protein coding genes that were differentially expressed from before to after HBO_2_ treatment in patients with NSTI. The functional profile of the differentially expressed gene set showed that upregulated genes were primarily involved in regulation of the T cell-driven immune response, and the downregulated genes were involved in inflammatory signaling and apoptosis.

### *Activation of T helper cells during treatment with HBO*_*2*_

Protein coding DEGs that were upregulated during HBO_2_ treatment were related to T helper cell signaling and differentiation and the adaptive immune system. All three transcription factors; GATA-3, T-bet and RORγ that govern the effector subsets of T helper cells; Th1, Th2 and Th17 were upregulated, along with upstream positive signaling transducers and activators as well as components of the T cell receptor. These key transcription factors possess dual action while both promoting one effector fate and repressing the alternative pathway in a complex transcription factor network (reviewed in [28]). This indicates that a polyclonal activation of CD4+ helper cells occurred within at least these three lineages following treatment with HBO_2_. In sepsis the number of CD4+ helper cells are greatly reduced following disease onset [29]. Research on the impact of HBO_2_ treatment on T cell activation and differentiation during severe infections is scant. In line with our findings, an experimental setup demonstrated polyclonal activation of T cells and a decrease in the production of pro-inflammatory cytokines in response to lipopolysaccharide (LPS) after HBO_2_ treatment [30]. Also, an in vitro model found that hyperbaric oxygen culture induced CD4+ regulatory cells in a Th2-type environment. [31]. In contrast, in healthy volunteers the level of CD4+ T cells decreased after a single exposure to HBO_2_ [32]. In the recent years it has been described how T helper cells adapt to changes in the microenvironment by forced expression of key regulators in differentiated T cells. This T helper cell plasticity is discovered to be highly integrated with tissue homeostasis and metabolic reprogramming, in particular changes in glucose metabolism [33]. This establishes a link to HBO_2_ treatment and provides a possible explanation for the effects described above; HBO_2_ treatment can cause changes in cellular energy metabolism, possibly causing lymphocytes to switch from glycolysis to oxidative phosphorylation [18, 34].

### *Downregulation of infection induced inflammatory signaling pathways during treatment with HBO*_*2*_

**Fig. 3:**
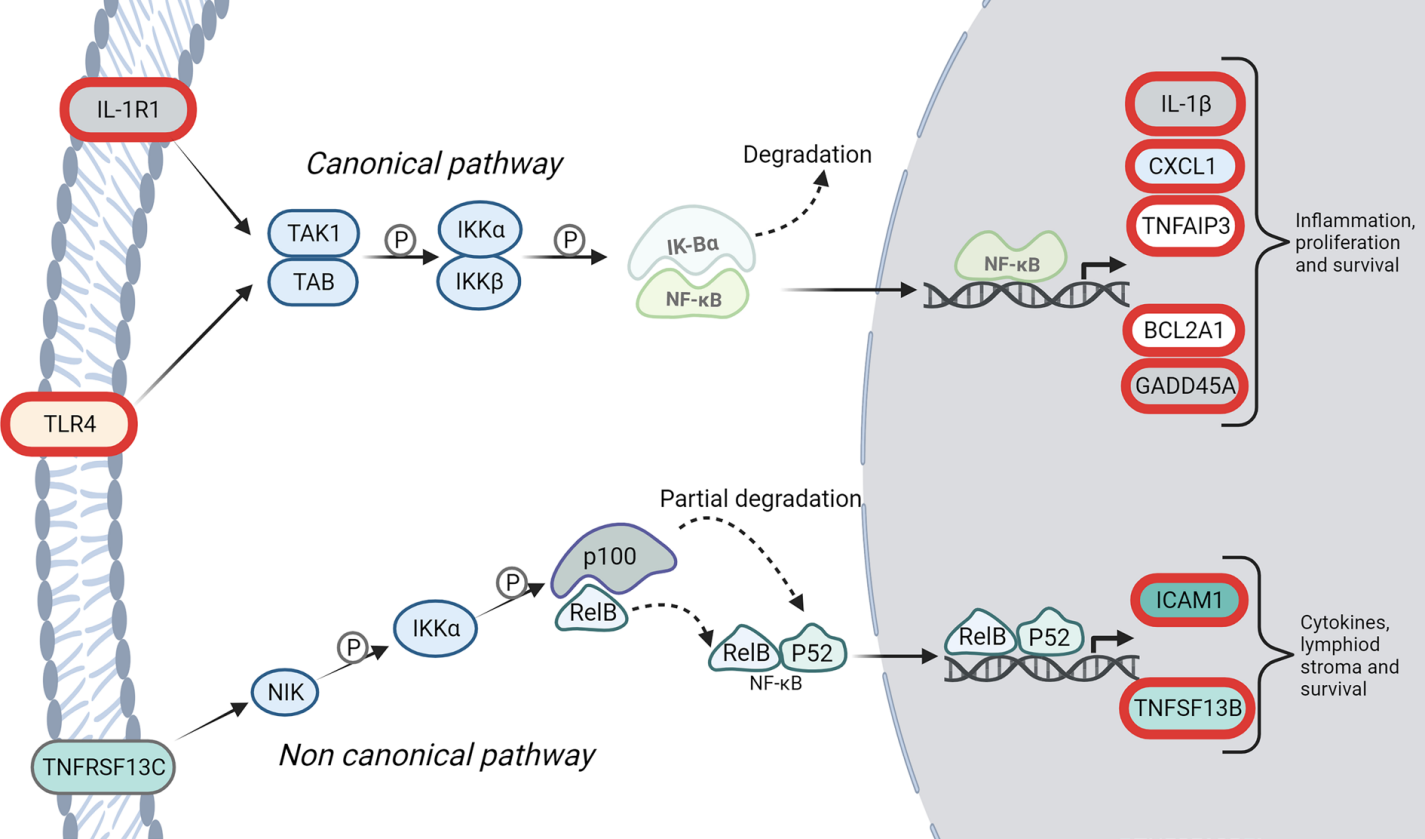
Illustration of the overrepresented genes in the KEGG pathway NF-κB signaling. Simplified version of the Kyoto Encyclopedia of Genes and Genomes (KEGG) pathway (hsa04064); NF-κB signaling pathway. Differentially expressed genes from immediately before to immediately after hyperbaric oxygen treatment that are significantly downregulated are marked in red (FDR < 0.02). Genes are named with their gene symbol

The nuclear factor-kappa B (NF-κB) signaling pathway was significantly downregulated during HBO_2_ treatment in our cohort of NSTI patients (Fig. 2B and Table 3). NF-κB is the generic name of a family of transcription factors that function as dimers and regulate genes involved in immunity, inflammation, and cell survival. Activation of the NF-κB pathway is known as the primary initiator of the hyperinflammatory cytokine storm in sepsis [19]. NF-κB is thought to be a redox-sensitive transcription factor and exposure to H_2_O_2_ or other oxidants has been shown to trigger nuclear translocation of NF-κB in certain cells, whereas antioxidants inhibit NF-κB induction in lymphoid cells (reviewed in [35]). The NF-κB pathway relies on IKK-mediated phosphorylation of IκBα (I-Kappa-B-Alpha), leading to its degradation. This allows NF-κB dimer to enter the cell nucleus and activate gene transcription (Fig. 3). IκBα mRNA expression is induced by LPS [36], and by NF-κB in an autoregulatory pathway due to a NF-κB responsive IκBα promoter [37], thus the downregulation of the expression of IκBα in the present study indicates a repression of NF-κB signaling. Interestingly, the ROS sensitive step in NF-κB activation is hypothesized to lie at the level of IκBα or immediately upstream thereof [35]. Downregulation of IκBα mRNA levels was accompanied by a reduction in the expression of several target genes, as well as the interleukin-1 receptor type 1 (IL-1R1) and Toll-like receptor 2 and 4 (TRL2- and 4); two important receptors for NF-kappa B-activation, which strongly promotes inflammation [38]. This is illustrated in Fig. 3 and results are available in Additional file 2: Table S4.

TLRs are expressed on all innate immune cells such as macrophages, neutrophils, dendritic cells, natural killer cells, mast cells, basophils, and eosinophils [39]. There is broad consensus that TLR2 and 4 cooperate at physiological concentrations of the ligands responsible for their activation, and it is believed that TLR4 is the receptor for LPS [40], whereas TLR2 is the receptor for Gram-positive bacterial cells [41]. The effect of HBO_2_ treatment on the mRNA expression of TLR2 and TLR4 has previously been investigated in an experimental rat model of zymosan-induced shock [42]. This study reported that HBO_2_ treatment downregulated the zymosan induced high levels of TLR2 and TLR4, and thereby specifically inhibited the translocation of NF-κB to the nucleus, which was associated to reduced organ dysfunction and amelioration of local and systemic inflammation [42]. Similar attenuating effects of HBO_2_ treatment on TLR4/NF-κB-mediated inflammation have been demonstrated in other models [43–45].

IL1R1 is the receptor for the two cytokines, IL-1α and IL-1β, of which IL-1β was also significantly downregulated in the present study (Fig. 3 and Table 3). IL-1β is a highly pro-inflammatory cytokine primarily transcribed by monocytes, macrophages, and dendritic cells following TLR activation by pathogen-associated molecular patterns or cytokine signaling [46]. NSTI patients have higher levels of IL-1β than patients with other cutaneous infections such as cellulitis [47]. The IL-1β network has also been identified as a critical network involved in the modulation of Group A streptococcal NSTIs in a rat model, and high levels of IL-1β were positively correlated with disease severity both in this model [48], and in a comparable cohort of NSTI patients [15]. Likewise, serum levels of IL-1β, IL-1-receptor antagonist (IL-1Ra), IL-18 and interferon-gamma has previously been demonstrated to be significantly elevated in NSTI patients with fatal outcome compared to survivors [49]. We found that both IL-1β and IL-1Ra mRNA expression, as well as their receptor IL1R1 and co-receptor IL1RAP (interleukin-1 receptor accessory protein) were significantly downregulated during HBO_2_ treatment, as were the IL-18 receptor IL18R1 and the accessory receptor IL18RAP (accessory receptors are not shown in the enrichment analysis). This indicates a downregulation of IL-1β signaling in our cohort of NSTI patients during treatment with HBO_2_. In an experimental setup, HBO_2_ treatment has previously been demonstrated to downregulate IL-1β production, both in a model of zymosan-induced multi-organ failure [42], in a model of LPS-induced IL-1β production in human blood-derived monocyte-macrophages [50], and in a rat model of endocarditis [51]. In contrast, the plasma protein expression of IL-1β was unchanged after 1 daily HBO_2_ session on 3 consecutive days in a comparable cohort of 209 NSTI patients [15]. This discrepancy between results on mRNA and protein expression in blood could either be explained by false discovery, post-transcriptional regulation of IL-1β mRNA, or if a reduced production of IL-1β is not reflected in the amount secreted to plasma. Interestingly, the secretion of mature IL-1β is accelerated in response to alteration in the cells basic redox state [52].

### *Downregulation of target genes involved in inflammation and apoptosis during treatment with HBO*_*2*_

Besides the above-mentioned downregulation of IL-1β and IL-18, the gene ontology overrepresentation analysis also displayed downregulation of the Arid5a (AT-rich interactive domain-containing protein 5a) mRNA (Fig. 3 and Table 3). TLR4-activated acetylation of the p65 subunit of NF-κB induces the transcriptional activation of the Arid5a promotor [53]. Upon inflammation, Arid5a translocates to the cytoplasm and stabilizes a variety of inflammatory mRNA transcripts and contributes to inflammatory responses, including septic shock [54].

Furthermore, several target genes involved in inflammation and regulatory apoptosis were displayed in the downregulated NF-κB pathway (Fig. 3). Regulation of TNFSF13B (Tumor Necrosis Factor Superfamily Member 13b), BCL2A1 (the B cell lymphoma protein 2 (Bcl-2) family member A1), TNFAIP3 (TNF alpha induced target gene 3) and GADD45A (Growth Arrest and DNA Damage Inducible Alpha) has not previously been linked to HBO_2_ treatment. However, mRNA expression of TNFSF13B, BCL2A1 and TNFAIP3 has been demonstrated to be redox sensitive. Hydrogen peroxide (H_2_O_2_) has been described to induce BCL2A1 gene expression in a T cell line, and this was described to occur in an NF-κB-dependent manner and was associated with cell survival [55]. Likewise, exogenous H_2_O_2_ treatment has been shown to increase TNFSF13B promoter activity, while activity was decreased by the ROS scavenger N-acetyl-cysteine [56]. The expression levels of TNFAIP3 have similarly been shown to be increasing in response to H_2_O_2_ and decreasing in response to N-acetyl cysteine, which was linked to increased endotoxin tolerance [57]. These four proteins are expressed by both adaptive and innate immune cells [58–61]. TNFSF13B [62], BCL2A1 [60] and GABB45α [58] are known to promote inflammation, and TNFSF13B [63] and GABB45α [58] have been described to be overexpressed in response to LPS, whereas both low and high levels of TNFAID3 have proven beneficial in severe infections [64, 65]. Particularly pertinent to NSTI is the discovery that group A streptococcus up-regulates TNFAIP3 in infected hosts to decrease the release of inflammatory cytokines, thereby facilitating immune surveillance escape and prolonging the pathogen's survival in the host [66].

ICAM-1 (intercellular adhesion molecule-1) expression has also been shown to be redox sensitive, as it is induced by both up-regulation of nitric oxide synthase [67] and exogenous H_2_O_2_ [68]. Furthermore, the therapeutic outcomes of HBO_2_ in patients with thermal burns have previously been linked to downregulation of ICAM-1 mRNA expression in blood [67]. This capacity of HBO_2_ treatment to reduce disease mediated elevated levels of ICAM-1 with subsequent amelioration of inflammation, has been verified in more models and conditions [69–71], with insignificant results in one study, however [72]. ICAM-1 is known to be increased in septic shock patients [73, 74], and models of experimental sepsis have demonstrated a less severe clinical course and improved survival rates in ICAM-1-knockout mice [75, 76]. Previous studies on the effect of HBO_2_ treatment on CXCL1 (C-X-C motif ligand (1) are limited to expression in astrocytes. In this cell type HBO_2_ also had ameliorating effects on high levels of CXCL1 following LPS stimulation or traumatic brain injury, which resulted in alleviated inflammation and injury [77, 78]. CXCL1 is a pro-inflammatory chemokine that is produced by macrophages, neutrophils, epithelial cells and Th17 cells, and in innate immune cells the expression of CXCL1 is dependent on IL-1β and TLR4 signaling. CXCL-1 acts as a chemoattractant for immune cells, especially neutrophils to the site of injury or infection [79]. The migration and infiltration of neutrophils to infectious sites during high CXCL1 expression aids in pathogen killing and has been shown to be critical in CXCL1-deficient animal models [79]. However, CXCL1 expression is also linked to many adverse conditions associated with uncontrolled inflammation and tissue damage, such as sepsis-associated encephalopathy, sepsis-associated acute kidney injury, and sepsis-induced lung injury [80]. Correspondingly, reducing LPS induced high levels of CXCL-1 has been associated with increased survival in animal models [81, 82].

### Strengths, limitations, and future perspectives

Some of the genes and pathways identified in this study have previously been linked to HBO_2_ cellular mechanisms of action, including NF-κB signaling, ICAM-1, IL-1ß and CXCL-1 [12, 42, 67, 78]. Besides that, like other studies investigating gene expression patterns in circulating lymphocytes [83], we identified immune response genes not previously described in the context, and Arid5a, TNFSF13B, and BCL2A1 are highlighted here as prospective future study topics. This demonstrates one advantage of using an omics systems approach rather than a potentially misguided hypothesis-driven approach, and as shown in Fig. 2, many genes and pathways were identified to be impacted by HBO_2_ treatment in this study, which we have not discussed further in the current paper. Although the mRNA level of the transcription factor NF-κB was not directly downregulated following HBO_2_ treatment, we may state that the current study indicated an overall downregulation of NF-κB signaling because of another equally obvious strength of the study design: the ability to examine the expression of a marker in the context of the pathway in which it operates. The used approach, however, also has some limitations. Enrichment analyses are susceptible to abundance biases, which occur because some genes have more annotations than the average gene. This could be genes that are strongly linked to specific diseases and thus receive more scientific attention and funding, or genes that are highly expressed genes and therefore easier to study; such biases can result in inflated significance and even artificial enrichment. In this regard, one advantage of the DAVID tool over other gene ontology analysis programs is that it uses annotation data from a broader range of sources. Another limitation of enrichment analysis is pathway overlap. Because the same genes can participate in multiple pathways, when cytokines are involved, pathways and terms involved in various inflammatory diseases can be identified, which also occurred in our study [see Fig. 2 and Additional File 2: Table S1–S4]. One limitation of studying gene expression is that it only provides information on mRNA expression. In the enrichment analysis, a highlighted transcript may never leave the nucleus in significant amounts as effective proteins. A previous study on the expression of ICAM-1 mRNA and protein levels in patients with injury-induced elevated levels of ICAM-1 in blood found a strong correlation [67]. This relationship between ICAM-1 gene and tissue expression was also demonstrated in an animal model, where HBO_2_ treatment also reduced injury-induced ICAM-1 expression [84]. BCL2A1 has also been described to be primarily transcriptionally regulated in human neutrophils isolated from peripheral blood [85], but for most of the described markers, various levels of post-transcriptional regulation may occur. In this regard it is important to note that in the present study we discovered a wide range of differentially expressed genes involved in immune functions, however all changes in gene expression level were of low fold change. This is to be expected given that the source of the differentially expressed mRNA was many cell populations, and thus large fold changes in a subpopulation will have been toned down. Even so, we were able to identify the most common immunological changes in lymphocytes that may be caused by HBO_2_ treatment intervention in this heterogeneous group of 85 highly dysregulated inflammatory patients by describing the larger cellular or physiological role carried out by the DEGs, coordinated with other DEGs through data analysis with overrepresentation analysis. Furthermore, even small changes in transcription factor mRNA expression levels can have dramatic biological effects, but effects on single effector proteins demonstrated in this study should be interpreted cautiously and in the context of the pathway in which they act. Another limitation of the study design is that the expression profile of all lymphoid cells is evaluated at the same time, which does not allow differentiation of which lymphoid subset a given gene is expressed in, nor does it allow evaluation of transitions of molecular components within the cell or transitions of lymphocyte subsets between peripheral blood and other lymphoid tissues or the injured target organ. A final challenge is related to the timing of sampling; while sampling close to the intervention is advantageous in terms of causality, it may have prematurely interrupted the signaling pathway, resulting in a lack of change at the target gene level in T helper cells in the enrichment analysis of the upregulated genes.

Nonetheless, because interventions are well defined and kept to a bare minimum due to the short time between samples, this study contributes to our understanding of the immunological mechanisms of action of HBO_2_ treatment. In the future, the findings of this paper should be confirmed by examining the presence of the highlighted DEGs post-transcriptionally, both in quantity and cellular location. This could be accomplished through single cell sequencing, or a multi-omics approach combining current transcriptomic data with proteomics and metabolomics, or through targeted protein panels combined with immunohistochemistry staining. The clinical relevance of the findings should be assessed by examining the relationship between HBO_2_-mediated downregulation of disease-induced elevated levels of inflammatory markers and clinical markers of disease severity.

## Conclusions

In summary, we identified and discussed genes and signaling pathways in whole blood that are regulated during HBO_2_ treatment in a cohort of 85 patients admitted to the intensive care unit with sepsis due to NSTI using enrichment analysis of bulk RNA sequencing data. When one or two sessions of HBO_2_ treatment are administered to these patients with a dysregulated immune response and systemic inflammation, we discovered that the important genes regulated during the intervention are involved in T helper cells activation and downregulation of the disease-induced highly inflammatory pathway NF-κB, which was associated with a decrease in the mRNA levels of the pro-inflammatory factors IL-1ß, ICAM-1, CXCL-1, TNFSF13B, Arid5a, and the cell-death regulating BCL2A1. We also found a downregulation in the expression of the genes GADD45α and TNFAIP3, both of which are known to be involved in the maintenance of cellular homeostasis in inflammatory conditions. Based on the present literature, it is probable that the identified alterations in immune cell gene expression will be connected to symptom alleviation in this cohort of highly inflammatory sepsis patients. However, the literature review reveals ambiguous results about potentially beneficial effect of TNFAIP3 downregulation in NSTI. Our review of the literature related to the affected target genes, also revealed that the NF-κB pathway, as well as four of the downregulated NF-κB target genes had previously been shown to be activated by high concentrations of H_2_O_2_, indicating that HBO_2_ does not raise, but rather lower, the levels of this reactive oxygen species in this cohort of patients with severe systemic infection.

### Supplementary Information

**Additional file 1: **A detailed description of the quality control performed, the data pre-processing including trimming and alignment, the differential expression analysis (**Fig. S1**) and the functional enrichment analysis.

**Additional file 2: **Additional results from quality control, differential expression analysis and the enrichment analyses using the GO -and KEGG databases. **Figure S2.** Histogram of p-values of differentially expressed genes. **Figure S3.** Volcano plot of differentially expressed genes. **Table S1.** Enriched GO biological processes from upregulated genes with FDR < 0.02. **Table S2**. Enriched KEGG pathways from upregulated genes with FDR < 0.02. **Table S3.** Enriched GO biological processes from downregulated genes with FDR < 0.02. **Table S4.** Enriched KEGG pathways from downregulated genes with FDR < 0.02. **Table S5.** Differentially expressed pro -and anti-inflammatory single markers with FDR < 0.05.

## Data Availability

The datasets generated and analyzed during the current study are not publicly available due to their sensitive nature, which precluded ethical authorities from approving data sharing without an approved personal data transfer agreement.

## Abbreviations

Arid5a: AT-rich interactive domain-containing protein 5a
BCL2: The B cell lymphoma protein 2
BCL2A1: The B cell lymphoma protein 2 (Bcl-2) family member A1
CXCL1: C-X-C motif ligand 1
DAVID: Database for Annotation, Visualization and Integrated Discovery
DEGs: Differentially expressed genes
FDR: False discovery rate
GADD45Α: Growth Arrest and DNA Damage Inducible Alpha
GATA-3: GATA-Binding Factor 3
GO: Gene Ontology
H2O2: Hydrogen peroxide
HBO2: Hyperbaric oxygen
ICAM-1: Intercellular adhesion molecule-1
ICU: Intensive care unit
IKK: Inhibitor of nuclear factor kappa-B kinase
IL-1: Interleukin 1
IL-10: Interleukin 10
IL-18: Interleukin 18
IL18R1: Interleukin receptor 18 type 1
IL18RAP: Interleukin-18 receptor accessory protein
IL-1R1: Interleukin-1 receptor type 1
IL-1Ra: IL-1-receptor antagonist
IL1RAP: Interleukin-1 receptor accessory protein
IL-1α: Interleukin 1-alpha
IL-1β: Interleukin 1-beta
IL-6: Interleukin 6
IL-8: Interleukin 8
IQR: Interquartile range
IκBα: I-Kappa-B-Alpha
KEGG: Kyoto Encyclopedia of Genes and Genomes
kPa: Kilo pascal
LPS: Lipopolysaccharide
NF-κB: Nuclear factor-kappa B
NSTI: Necrotizing soft tissue infection
p65: Nuclear Factor NF-Kappa-B P65 Subunit
RORγ: Retinoic-related orphan receptors-Gamma
T-bet: T-Box Transcription Factor 21
TGF-β: Tumor growth factor-beta
TIN: Transcript integrity score
TNFAIP3: TNF alpha induced target gene 3
TNFSF13B: Tumor Necrosis Factor Superfamily Member 13b
TRL2: Toll-like receptor 2
TRL4: Toll-like receptor 4

## References

[1] Hedetoft M, Madsen MB, Madsen LB, Hyldegaard O (2020). Incidence, comorbidity and mortality in patients with necrotising soft-tissue infections, 2005–2018: a Danish nationwide register-based cohort study. BMJ Open.

[2] Das DK, Baker MG, Venugopal K (2011). Increasing incidence of necrotizing fasciitis in New Zealand: a nationwide study over the period 1990 to 2006. J Infect.

[3] Madsen MB, Skrede S, Perner A, Arnell P, Nekludov M, Bruun T (2019). Patient's characteristics and outcomes in necrotising soft-tissue infections: results from a Scandinavian, multicentre, prospective cohort study. Intensive Care Med.

[4] Singer M, Deutschman CS, Seymour CW, Shankar-Hari M, Annane D, Bauer M (2016). The third international consensus definitions for sepsis and septic shock (Sepsis-3). JAMA.

[5] Wiersinga WJ, van der Poll T (2022). Immunopathophysiology of human sepsis. EBioMedicine.

[6] Levett D, Bennett MH, Millar I (2015). Adjunctive hyperbaric oxygen for necrotizing fasciitis. Cochrane Database Syst Rev.

[7] Moon RE, Bakker D, Barnes R, Bennett M, Camporesi E, Cianci P (2019). Hyperbaric oxygen therapy indications.

[8] Schottlender N, Gottfried I, Ashery U (2021). Hyperbaric oxygen treatment: effects on mitochondrial function and oxidative stress. Biomolecules.

[9] Brummelkamp WH (1966). Treatment of infections due to anaerobic germs by inhalation of hyperbaric oxygen. Ann Chir Thorac Cardiovasc.

[10] Amlbomc C (1980). L’oxygdnoth6rapie hyperbare (O.H.B.) dans le traitement des toxi-infections a germes anadrobies. Med Mal Infect.

[11] Hadanny A, Efrati S (2020). The hyperoxic-hypoxic paradox. Biomolecules.

[12] De Wolde SD, Hulskes RH, Weenink RP, Hollmann MW, Van Hulst RA (2021). The effects of hyperbaric oxygenation on oxidative stress inflammation and angiogenesis. Biomolecules.

[13] Kot J, Lenkiewicz E (2022). Hyperbaric oxygen therapy in necrotizing soft tissue infections caused by Vibrio species from the Baltic Sea—three clinical cases. Int Marit Health.

[14] Hedetoft M, Moser C, Jensen PO, Vinkel J, Hyldegaard O (1985). Soluble ICAM-1 is modulated by hyperbaric oxygen treatment and correlates with disease severity and mortality in patients with necrotizing soft-tissue infection. J Appl Physiol.

[15] Hedetoft M, Garred P, Madsen MB, Hyldegaard O (2021). Hyperbaric oxygen treatment is associated with a decrease in cytokine levels in patients with necrotizing soft-tissue infection. Physiol Rep.

[16] Hedetoft M, Jensen PO, Moser C, Vinkel J, Hyldegaard O (2021). Hyperbaric oxygen treatment impacts oxidative stress markers in patients with necrotizing soft-tissue infection. J Investig Med.

[17] Buras JA, Holt D, Orlow D, Belikoff B, Pavlides S, Reenstra WR (2006). Hyperbaric oxygen protects from sepsis mortality via an interleukin-10-dependent mechanism. Crit Care Med.

[18] Vinkel J, Arenkiel B, Hyldegaard O (2023). The mechanisms of action of hyperbaric oxygen in restoring host homeostasis during sepsis. Biomolecules.

[19] Arora J, Mendelson AA, Fox-Robichaud A (2023). Sepsis: network pathophysiology and implications for early diagnosis. Am J Physiol-Regul, Integr Comp Physiol.

[20] Vinkel J, Rib L, Buil A, Hedetoft M, Hyldegaard O (2022). Investigating the effects of hyperbaric oxygen treatment in necrotizing soft tissue infection with transcriptomics and machine learning (the HBOmic study): protocol for a prospective cohort study with data validation. JMIR Res Protoc.

[21] Dobin A, Davis CA, Schlesinger F, Drenkow J, Zaleski C, Jha S (2013). STAR: ultrafast universal RNA-seq aligner. Bioinformatics.

[22] Robinson MD, McCarthy DJ, Smyth GK (2010). edgeR: a Bioconductor package for differential expression analysis of digital gene expression data. Bioinformatics.

[23] Ashburner M, Ball CA, Blake JA, Botstein D, Butler H, Cherry JM (2000). Gene ontology: tool for the unification of biology. The gene ontology consortium. Nat Genet.

[24] Kanehisa M, Goto S (2000). KEGG: kyoto encyclopedia of genes and genomes. Nucleic Acids Res.

[25] Dennis G, Sherman BT, Hosack DA, Yang J, Gao W, Lane HC (2003). DAVID: database for annotation, visualization, and integrated discovery. Genome Biol.

[26] Walter W, Sanchez-Cabo F, Ricote M (2015). GOplot: an R package for visually combining expression data with functional analysis. Bioinformatics.

[27] Ge X, Jung D, Yao R (2019). ShinyGO: a graphical enrichment tool for animals and plants. Bioinformatics.

[28] Capone A, Volpe E (2020). Transcriptional regulators of T helper 17 Cell differentiation in health and autoimmune diseases. Front Immunol.

[29] Chen X, Ye J, Ye J (2011). Analysis of peripheral blood lymphocyte subsets and prognosis in patients with septic shock. Microbiol Immunol.

[30] Kudchodkar B, Jones H, Simecka J, Dory L (2008). Hyperbaric oxygen treatment attenuates the pro-inflammatory and immune responses in apolipoprotein E knockout mice. Clin Immunol.

[31] MacKenzie DA, Sollinger HW, Hullett DA (2000). Role of CD4+ regulatory T cells in hyperbaric oxygen-mediated immune nonresponsiveness. Hum Immunol.

[32] Bitterman N, Bitterman H, Kinarty A, Melamed Y, Lahat N (1993). Effect of a single exposure to hyperbaric oxygen on blood mononuclear cells in human subjects. Undersea Hyperb Med.

[33] Saravia J, Chapman NM, Chi H (2019). Helper T cell differentiation. Cell Mol Immunol.

[34] Tezgin D, Giardina C, Perdrizet GA, Hightower LE (2020). The effect of hyperbaric oxygen on mitochondrial and glycolytic energy metabolism: the caloristasis concept. Cell Stress Chaperones.

[35] Bubici C, Papa S, Dean K, Franzoso G (2006). Mutual cross-talk between reactive oxygen species and nuclear factor-kappa B: molecular basis and biological significance. Oncogene.

[36] Chen Y, Zheng Y, Zhou Z, Wang J (2018). Baicalein alleviates tubular-interstitial nephritis in vivo and in vitro by down-regulating NF-kappaB and MAPK pathways. Braz J Med Biol Res.

[37] Werner SL, Kearns JD, Zadorozhnaya V, Lynch C, O'Dea E, Boldin MP (2008). Encoding NF-kappaB temporal control in response to TNF: distinct roles for the negative regulators IkappaBalpha and A20. Genes Dev.

[38] Dinarello CA (2011). Interleukin-1 in the pathogenesis and treatment of inflammatory diseases. Blood.

[39] Akira S (2001). Toll-like receptors and innate immunity. Adv Immunol.

[40] Beutler B, Du X, Poltorak A (2001). Identification of Toll-like receptor 4 (Tlr4) as the sole conduit for LPS signal transduction: genetic and evolutionary studies. J Endotoxin Res.

[41] Yoshimura A, Lien E, Ingalls RR, Tuomanen E, Dziarski R, Golenbock D (1999). Cutting edge: recognition of gram-positive bacterial cell wall components by the innate immune system occurs via Toll-like receptor 2. J Immunol.

[42] Rinaldi B, Cuzzocrea S, Donniacuo M, Capuano A, Di Palma D, Imperatore F (2011). Hyperbaric oxygen therapy reduces the toll-like receptor signaling pathway in multiple organ failures. Intensive Care Med.

[43] Kang N, Hai Y, Yang J, Liang F, Gao CJ (2015). Hyperbaric oxygen intervention reduces secondary spinal cord injury in rats via regulation of HMGB1/TLR4/NF-kappaB signaling pathway. Int J Clin Exp Pathol.

[44] Meng XE, Zhang Y, Li N, Fan DF, Yang C, Li H (2016). Hyperbaric oxygen alleviates secondary brain injury after trauma through inhibition of TLR4/NF-kappaB signaling pathway. Med Sci Monit.

[45] Liu XH, Liang F, Jia XY, Zhao L, Zhou Y, Yang J (2020). Hyperbaric oxygen treatment improves hearing level via attenuating TLR4/NF-kappaB mediated inflammation in sudden sensorineural hearing loss patients. Biomed Environ Sci.

[46] Fields JK, Gunther S, Sundberg EJ (2019). Structural basis of IL-1 family cytokine signaling. Front Immunol.

[47] Rath E, Palma Medina LM, Jahagirdar S, Mosevoll KA, Damås JK, Madsen MB (2023). Systemic immune activation profiles in streptococcal necrotizing soft tissue infections: a prospective multicenter study. Clin Immunol.

[48] Nookala S, Krishnan KC, Mukundan S, Kotb M (2020). Systems genetics approaches in mouse models of group a streptococcal necrotizing soft-tissue infections. Adv Exp Med Biol.

[49] Lungstras-Bufler K, Bufler P, Abdullah R, Rutherford C, Endres S, Abraham E (2004). High cytokine levels at admission are associated with fatal outcome in patients with necrotizing fasciitis. Eur Cytokine Netw.

[50] Benson RM, Minter LM, Osborne BA, Granowitz EV (2003). Hyperbaric oxygen inhibits stimulus-induced proinflammatory cytokine synthesis by human blood-derived monocyte-macrophages. Clin Exp Immunol.

[51] Lerche CJ, Christophersen LJ, Kolpen M, Nielsen PR, Trostrup H, Thomsen K (2017). Hyperbaric oxygen therapy augments tobramycin efficacy in experimental Staphylococcus aureus endocarditis. Int J Antimicrob Agents.

[52] Tassi S, Carta S, Delfino L, Caorsi R, Martini A, Gattorno M (2010). Altered redox state of monocytes from cryopyrin-associated periodic syndromes causes accelerated IL-1beta secretion. Proc Natl Acad Sci U S A.

[53] Nyati KK, Masuda K, Zaman MM, Dubey PK, Millrine D, Chalise JP (2017). TLR4-induced NF-kappaB and MAPK signaling regulate the IL-6 mRNA stabilizing protein Arid5a. Nucleic Acids Res.

[54] Nyati KK, Agarwal RG, Sharma P, Kishimoto T (2019). Arid5a regulation and the roles of Arid5a in the inflammatory response and disease. Front Immunol.

[55] Kim H, Kim YN, Kim H, Kim CW (2005). Oxidative stress attenuates Fas-mediated apoptosis in Jurkat T cell line through Bfl-1 induction. Oncogene.

[56] Lee GH, Lee MH, Yoon YD, Kang JS, Pyo S, Moon EY (2012). Protein kinase C stimulates human B cell activating factor gene expression through reactive oxygen species-dependent c-Fos in THP-1 pro-monocytic cells. Cytokine.

[57] Li Y, Zhang P, Wang C, Han C, Meng J, Liu X (2013). Immune responsive gene 1 (IRG1) promotes endotoxin tolerance by increasing A20 expression in macrophages through reactive oxygen species. J Biol Chem.

[58] Schmitz I (2022). Gadd45 proteins in immunity 2.0. Adv Exp Med Biol.

[59] Harhaj EW, Dixit VM (2012). Regulation of NF-kappaB by deubiquitinases. Immunol Rev.

[60] Vogler M (2012). BCL2A1: the underdog in the BCL2 family. Cell Death Differ.

[61] Huard B, Schneider P, Mauri D, Tschopp J, French LE (2001). T cell costimulation by the TNF ligand BAFF. J Immunol.

[62] Sutherland AP, Ng LG, Fletcher CA, Shum B, Newton RA, Grey ST (2005). BAFF augments certain Th1-associated inflammatory responses. J Immunol.

[63] Brinkhoff A, Zeng Y, Sieberichs A, Dolff S, Shilei X, Sun M (2019). Biosci Rep.

[64] Kang DR, Yoon GY, Cho J, Lee SJ, Lee SJ, Park HJ (2017). Neoagarooligosaccharides prevent septic shock by modulating A20-and cyclooxygenase-2-mediated interleukin-10 secretion in a septic-shock mouse model. Biochem Biophys Res Commun.

[65] Martin-Vicente M, Gonzalez-Sanz R, Cuesta I, Monzon S, Resino S, Martinez I (2020). Downregulation of A20 expression increases the immune response and apoptosis and reduces virus production in cells infected by the human respiratory syncytial virus. Vaccines.

[66] Ma C, Gao X, Wu S, Zhang L, Wang J, Zhang Z (2018). M protein of group a streptococcus plays an essential role in inducing high expression of A20 in macrophages resulting in the downregulation of inflammatory response in lung tissue. Front Cell Infect Microbiol.

[67] Oley MH, Oley MC, Aling DMR, Kalangi JA, Islam AA, Hatta M (2021). Effects of hyperbaric oxygen therapy on the healing of thermal burns and its relationship with ICAM-1: a case-control study. Ann Med Surg.

[68] Roebuck KA (1999). Oxidant stress regulation of IL-8 and ICAM-1 gene expression: differential activation and binding of the transcription factors AP-1 and NF-kappaB (Review). Int J Mol Med.

[69] Song K, Zhang M, Liu Y, Wang Y, Ma X (2016). The effect of hyperbaric oxygen preconditioning on the expression of ICAM-1, VCAM-1, NF-κB and flap survival rate during ischemia-reperfusion injury in rat abdominal skin flap. Zhonghua Zheng Xing Wai Ke Za Zhi.

[70] Khiabani KT, Bellister SA, Skaggs SS, Stephenson LL, Nataraj C, Wang WZ (2008). Reperfusion-induced neutrophil CD18 polarization: effect of hyperbaric oxygen. J Surg Res.

[71] Liu H, Yang M, Pan L, Liu P, Ma L (2016). Hyperbaric oxygen intervention modulates early brain injury after experimental subarachnoid hemorrhage in rats: possible involvement of TLR4/NF-x03BA B-mediated signaling pathway. Cell Physiol Biochem.

[72] Demirci H, Polat Z, Uygun A, Kadayifci A, Sager O, Oztutk K (2016). The effect of the iNOS inhibitor S-methylisothiourea and hyperbaric oxygen treatment on radiation colitis in rats. Acta Gastroenterol Belg.

[73] Sessler CN, Windsor AC, Schwartz M, Watson L, Fisher BJ, Sugerman HJ (1995). Circulating ICAM-1 is increased in septic shock. Am J Respir Crit Care Med.

[74] Linda Bara JE, Oss P, Cauce V, Hagina E, Gintere S, Viksna L, Krumina A (2019). Development of sepsis in relation to serum biomarkers concentration like intercellular adhesion molecule-1, macrophage migration inhibitory factor, plasminogen activator inhibitor-1 and soluble Fas ligand. Clin Pract.

[75] Hildebrand F, Pape HC, Harwood P, Muller K, Hoevel P, Putz C (2005). Role of adhesion molecule ICAM in the pathogenesis of polymicrobial sepsis. Exp Toxicol Pathol.

[76] van Griensven M, Probst C, Muller K, Hoevel P, Pape HC (2006). Leukocyte-endothelial interactions via ICAM-1 are detrimental in polymicrobial sepsis. Shock.

[77] Xia A, Huang H, You W, Liu Y, Wu H, Liu S (2022). The neuroprotection of hyperbaric oxygen therapy against traumatic brain injury via NF-κB/MAPKs-CXCL1 signaling pathways. Exp Brain Res.

[78] Liu S, Lu C, Liu Y, Zhou X, Sun L, Gu Q (2018). Hyperbaric oxygen alleviates the inflammatory response induced by LPS through inhibition of NF-kappaB/MAPKs-CCL2/CXCL1 signaling pathway in cultured astrocytes. Inflammation.

[79] Biondo C, Mancuso G, Midiri A, Signorino G, Domina M, Lanza Cariccio V (2014). The interleukin-1β/CXCL1/2/neutrophil axis mediates host protection against group B streptococcal infection. Infect Immun.

[80] Korbecki J, Maruszewska A, Bosiacki M, Chlubek D, Baranowska-Bosiacka I (2022). The potential importance of CXCL1 in the physiological state and in noncancer diseases of the cardiovascular system, respiratory system and skin. Int J Mol Sci.

[81] Kim C, Sim H, Bae JS (2022). Benzoylpaeoniflorin activates anti-inflammatory mechanisms to mitigate sepsis in cell-culture and mouse sepsis models. Int J Mol Sci.

[82] Ahn JH, Song EJ, Jung DH, Kim YJ, Seo IS, Park SC (2022). The sesquiterpene lactone estafiatin exerts anti-inflammatory effects on macrophages and protects mice from sepsis induced by LPS and cecal ligation puncture. Phytomedicine.

[83] Wang M, Windgassen D, Papoutsakis ET (2008). Comparative analysis of transcriptional profiling of CD3+, CD4+ and CD8+ T cells identifies novel immune response players in T-cell activation. BMC Genomics.

[84] Lavrnja I, Parabucki A, Brkic P, Jovanovic T, Dacic S, Savic D (2015). Repetitive hyperbaric oxygenation attenuates reactive astrogliosis and suppresses expression of inflammatory mediators in the rat model of brain injury. Mediators Inflamm.

[85] Vier J, Groth M, Sochalska M, Kirschnek S (2016). The anti-apoptotic Bcl-2 family protein A1/Bfl-1 regulates neutrophil survival and homeostasis and is controlled via PI3K and JAK/STAT signaling. Cell Death Dis.

[86] Madsen MB, Skrede S, Bruun T, Arnell P, Rosen A, Nekludov M (2018). Necrotizing soft tissue infections—a multicentre, prospective observational study (INFECT): protocol and statistical analysis plan. Acta Anaesthesiol Scand.

