# Supplementary material for: Key pathways and genes that are altered during treatment with hyperbaric oxygen in patients with sepsis due to necrotizing soft tissue infection (HBOmic study)
Source: Eur J Med Res. 2023 Nov 10;28:507. doi: 10.1186/s40001-023-01466-z (PMC10636866; doi:10.1186/s40001-023-01466-z)
Supplement: Supplementary file 1 — Additional file 1: A detailed description of the quality control performed, the data pre-processing including trimming and alignment, the differential expression analysis (Fig. S1) and the functional enrichment analysis. [file 40001_2023_1466_MOESM1_ESM.docx]

**Additional file 1, Supplementary methods**

*Quality control*

Quality control of sequence reads was performed using the tools: 1) “FastQC” v0.11.7 (http://www.bioinformatics.babraham.ac.uk/projects/fastqc/) was used to check sequence counts, sequence quality, per base contents and read length distributions. 2) “fastq_screen” v0.11.4 (https://www.bioinformatics.babraham.ac.uk/projects/fastq_screen/) was used to check whether samples were contaminated. 3) “RSeQC” v2.6.4 [1] was used to calculate Transcript integrity numbers (TIN) [2] and read gene body coverage. All the quality information was combined using the tool MultiQC. For downstream steps, only samples with a TIN score higher than 50 (out of 100) were used (this discarded 4 samples).

*Data pre-processing*

The first 14 bases and reads shorter than 35 nt were removed with “Trimmomatic” v0.39 [3] using the settings “HEADCROP:14 LEADING:3 SLIDINGWINDOW:4:15 MINLEN:35”. Reads were mapped using “STAR” 2.7.3a [4] against the human genome (hg38). Up to two mismatches were allowed during the mapping, and the minimum number of overlap bases to trigger mates merging and realignment was set to five. Otherwise, default settings were used. Duplicate reads were also removed with the bamRemoveDuplicatesType "UniqueIdentical" option in “STAR”. The “featureCounts” function of the “Rsubread” R package v2.2.6 [5] was used to quantify reads in exons. The Gencode v38 comprehensive gene annotation including all genomic regions was used to assign reads to genes.

*Differential expression*

The “edgeR” v3.30.3 [6] R package was used to perform a differential expression analysis comparing samples before vs after HBO_2_ treatment. For this purpose, first a model was defined indicating the sampling time and the patient identifier to set a paired analysis. Library normalization factors were calculated using the “calcNormFactors” function with the “TMM” algorithm. Tag-wise dispersion was calculated using the “estimateDisp” function with “robust = TRUE”. A gene-wise generalized linear model was fit with “glmQLFit”, using the following covariates: patientId and sampling, where sampling is whether the transcript belongs to the group before or after hyperbaric oxygen treatment, figure S4

Fig. A1 Generalized linear model applied for differential expression

fit <- glmQLfit (patientId + sampling)

Finally, differential gene usage was assessed using “glmQLFTest”. Resulting p-values were corrected for multiple testing using the Benjamini–Hochberg method. Genes were considered differentially expressed if they had a P value lower than 0.05 and a log2 fold-change across conditions higher than 1.

*Annotation of differentially expressed genes*

Functional enrichment analysis was performed using Gene Ontology (GO) and Kyoto Encyclopedia of Genes and Genomes (KEGG) pathway enrichment analyses as described in the main paper. For the main analyses as presented in the main paper we included only genes that were differentially expressed with a significance level of FDR < 0.01 to minimize the risk of including false positives in the differential expression analysis. As these are arbitrary choices, we decided to complement these primarily results with the result from the GO enrichment analysis of all genes that were differentially expressed with a significance level of FDR < 0.02, as presented in the supplementary result section.

**References for supplementary methods**

1. Wang L, Wang S, Li W. RSeQC: quality control of RNA-seq experiments. Bioinformatics*.* 2012;28:2184-2185. DOI: 10.1093/bioinformatics/bts356.

2. Wang L, Nie J, Sicotte H, Li Y, Eckel-Passow JE, Dasari S, et al. Measure transcript integrity using RNA-seq data. BMC Bioinformatics*.* 2016;17:58. DOI: 10.1186/s12859-016-0922-z.

3. Bolger AM, Lohse M, Usadel B. Trimmomatic: a flexible trimmer for Illumina sequence data. Bioinformatics*.* 2014;30:2114-2120. DOI: 10.1093/bioinformatics/btu170.

4. Dobin A, Davis CA, Schlesinger F, Drenkow J, Zaleski C, Jha S, et al. STAR: ultrafast universal RNA-seq aligner. Bioinformatics*.* 2013;29:15-21. DOI: 10.1093/bioinformatics/bts635.

5. Liao Y, Smyth GK, Shi W. The R package Rsubread is easier, faster, cheaper and better for alignment and quantification of RNA sequencing reads. Nucleic Acids Res*.* 2019;47. DOI: 10.1093/nar/gkz114.

6. Robinson MD, McCarthy DJ, Smyth GK. edgeR: a Bioconductor package for differential expression analysis of digital gene expression data. Bioinformatics*.* 2010;26:139-140. DOI: 10.1093/bioinformatics/btp616.
