# Supplementary material for: Key pathways and genes that are altered during treatment with hyperbaric oxygen in patients with sepsis due to necrotizing soft tissue infection (HBOmic study)
Source: Eur J Med Res. 2023 Nov 10;28:507. doi: 10.1186/s40001-023-01466-z (PMC10636866; doi:10.1186/s40001-023-01466-z)
Supplement: Supplementary file 2 — Additional file 2: Additional results from quality control, differential expression analysis and the enrichment analyses using the GO -and KEGG databases. Figure S2. Histogram of p-values of differentially expressed genes. Figure S3. Volcano plot of differentially expressed genes. Table S1. Enriched GO biological processes from upregulated genes with FDR < 0.02. Table S2. Enriched KEGG pathways from upregulated genes with FDR < 0.02. Table S3. Enriched GO biological processes from downregulated genes with FDR < 0.02. Table S4. Enriched KEGG pathways from downregulated genes with FDR < 0.02. Table S5. Differentially expressed pro -and anti-inflammatory single markers with FDR < 0.05. [file 40001_2023_1466_MOESM2_ESM.docx]

**Additional file 2, Supplementary results**

*Quality control*

The sample quality control of the extracted RNA form whole blood cells revealed a mean RNA quality number of 8.77 (SD 1.57), and a median of 2493 (IQR 2627.25) ng total RNA extracted from the blood samples.

The statistics generated by the Multiqc report of the reads mapped to the human genome revealed an average of 49.943.347 reads per sample, with a minimum of 17.070.250 and

a maximum of 135.041.966 reads. All the reads were of good quality on average (Phred score > 30), in all 130 base calls across the read. We obtained an average of 11872330.65 unique reads with an average of 15487650.72 duplicates. After cleaning for duplicates, we ended up with an average of 39.891.865 reads per sample. We did not remove any samples based on the MultiQC.

*Differential expression*

For the differential expression analysis, we chose to exclude 4 samples with a TIN < 0.5. The remaining 160 samples, with a mean TIN score of 69.28 (+/- 4.8) proceeded to differential expression analysis.

Fig. A2 Histogram of p-values of differentially expressed genes


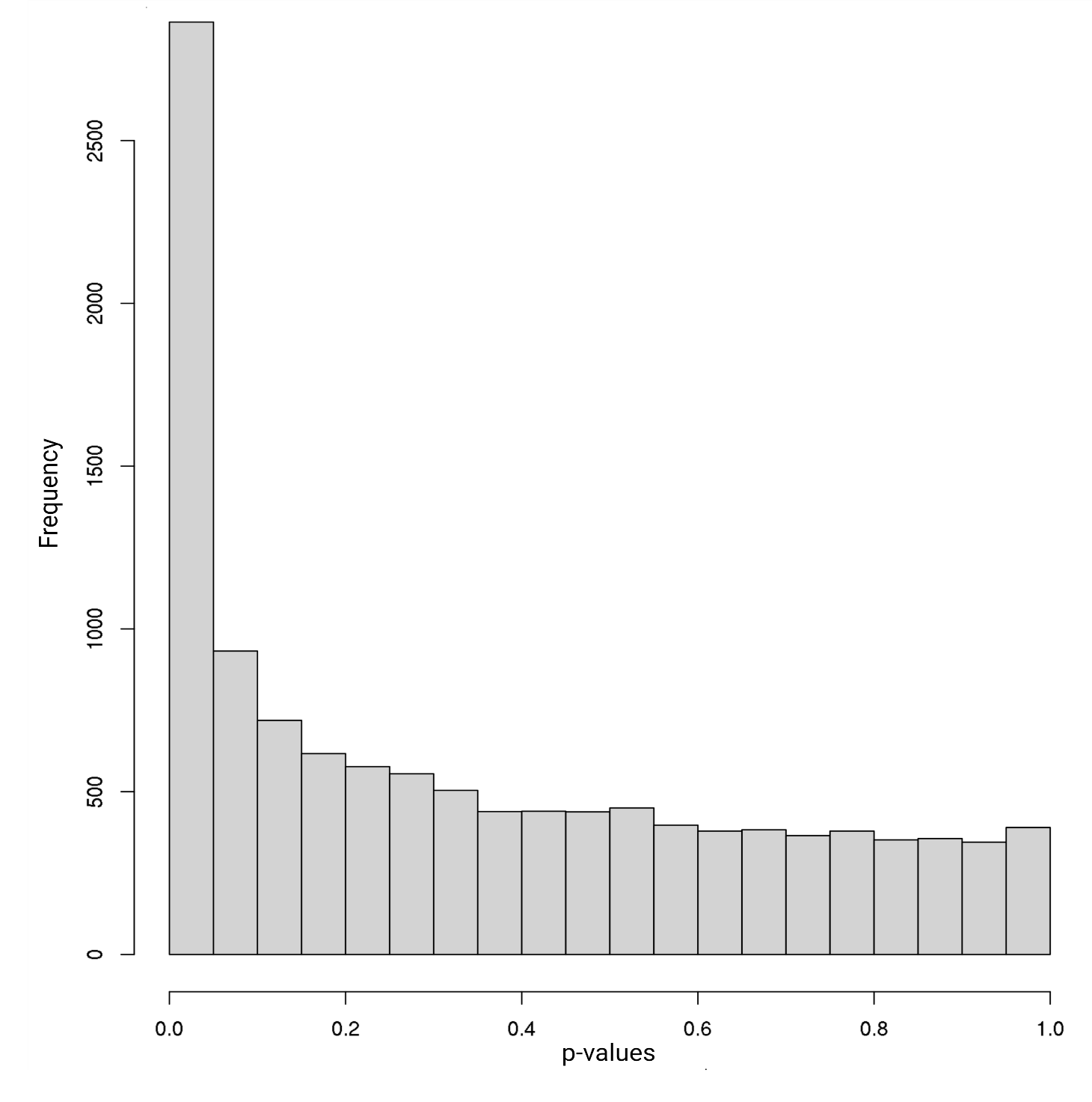


Histogram of p-values from the differential expression analysis. The x-axis shows the p-values, and the y-axis shows the frequency of those p-values. Only genes with a logCPM > 0 were used to make the plot.

Fig. A3 Volcano plot of differentially expressed genes


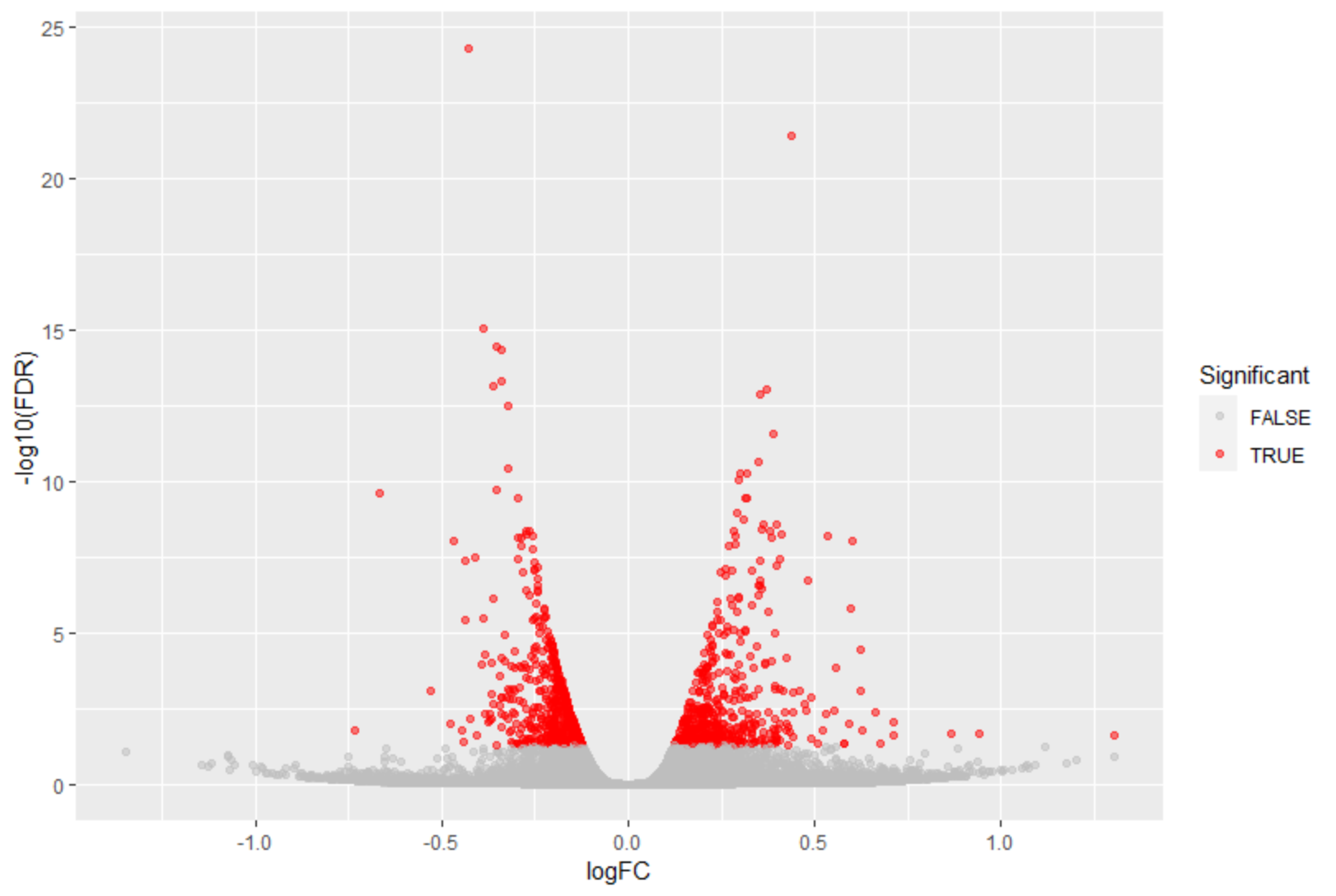


Volcanoplot of significance (-log10(FDR) plotted against foldchange (logFC). Significant differentially expressed genes (FDR<0.05) are marked with red. The most significant downregulated gene is RLF (Zinc Finger Protein Rlf), and the most significant upregulated gene is DEFA3 (Defensin Alpha 3). The gene products with the highest absolute fold change are the non-coding RNA products; BX255923.1 (upregulated) and AL355981.1 (downregulated).

Table A1 Enriched GO biological processes from upregulated genes

| Term | Name | % | Genes | Fold Enrichment | FDR |
| --- | --- | --- | --- | --- | --- |
| GO:0002250 | adaptive immune response | 9.4 | ITK, TXK, CD3G, CD3E, CD3D, JCHAIN, KLRC1, EOMES, ZNF683, LAG3, BTN3A1, TRAT1, SH2D1A, THEMIS, SH2D1B, LAX1, ZAP70, CD6, CD8B, CD8A, CAMK4, TARM1, CD7, KLRD1, CD247, SKAP1 | 8.6 | 5.16E-13 |
| GO:0050852 | T cell receptor signaling pathway | 6.9 | ZNF683, ITK, BTN3A1, TXK, RFTN1, THEMIS, BTN3A3, CD3G, GATA3, CD3E, CD3D, ZAP70, CD8B, LCK, CD8A, CD28, PLCG1, CD247, SKAP1 | 12.0 | 1.66E-11 |
| GO:0007166 | cell surface receptor signaling pathway | 9.1 | MS4A3, KLRB1, CD3G, CD3E, CD3D, SPN, ADGRG1, ADGRG5, ERBB2, MS4A14, KLRC1, KLRG1, LAG3, FCRL6, FCRL3, CD2, CD8A, KLRF1, CD28, EVL, TNFRSF25, KLRD1, CD247, IL7R, MS4A1 | 6.6 | 2.74E-10 |
| GO:0042110 | T cell activation | 5.1 | ITK, LAG3, NLRC3, CD3G, CD3E, RASGRP1, LY9, DPP4, CD2, ZAP70, CD8B, CD8A, CD7, CD28 | 17.3 | 3.25E-10 |
| GO:0006955 | immune response | 8.3 | IL32, CD96, GZMA, KIR3DL1, FASLG, CTSW, LAX1, JCHAIN, SPN, TGFBR3, ZAP70, CYSLTR2, TMIGD2, CNR2, CD8B, RGS1, CD8A, CD7, CTSG, CEACAM8, IL7R, TNFRSF4, C1QC | 4.3 | 6.79E-06 |
| GO:0006968 | cellular defense response | 2.9 | SPN, ITK, FCMR, GNLY, TRAT1, SH2D1A, PRF1, KLRG1 | 9.9 | 0.0039 |
| GO:0031295 | T cell costimulation | 2.5 | DPP4, TMIGD2, CD5, LCK, CD28, CD3E, CARD11 | 11.2 | 0.0071 |
| GO:0046631 | alpha-beta T cell activation | 1.4 | CD3G, CD247, CD3E, CD3D | 51.4 | 0.0071 |
| GO:0050832 | defense response to fungus | 2.2 | SPON2, GNLY, DEFA4, DEFA3, CTSG, MPO | 11.3 | 0.0279 |
| GO:0019731 | antibacterial humoral response | 2.5 | DEFA4, DEFA3, CTSG, PI3, RNASE3, AZU1, JCHAIN | 8.3 | 0.0279 |
| GO:0046629 | gamma-delta T cell activation | 1.4 | ITK, CD3G, CD247, CD3E | 32.1 | 0.0279 |
| GO:0019835 | cytolysis | 1.8 | GZMM, GZMA, PRF1, GZMB, GZMH | 14.0 | 0.0521 |

Results from gene enrichment analysis with Gene ontology (GO). All differentially expressed genes with a cut off FDR < 0.02 and positive fold change were included in the analysis. Analysis is performed with the DAVID bioinformatic tool. Term and name are the enriched GO enriched biologic process using the DAVID default function BP_DIRECT, sorted by significance. % is the percentage of differentially expressed genes belonging to the pathway. Genes are the gene symbol of the differentially expressed genes belonging to the pathway. Fold enrichment is the percentage of differentially expressed genes belonging to the pathway, divided by the corresponding percentage in the background. FDR is the significance level after correction for multiple testing with false discovery rate.

Table A2 Enriched KEGG pathways from upregulated genes

| Term | Name | % | Genes | Fold Enrichment | FDR |
| --- | --- | --- | --- | --- | --- |
| hsa04660 | T cell receptor signaling pathway | 5.1 | ITK, NFATC2, CD3G, CD3E, RASGRP1, CD3D, ZAP70, CD8B, LCK, CD8A, CD28, PLCG1, CD247, CARD11 | 7.7 | 4.55E-06 |
| hsa04658 | Th1 and Th2 cell differentiation | 4.7 | NFATC2, CD3G, GATA3, CD3E, RUNX3, CD3D, ZAP70, LCK, TBX21, IL2RB, STAT4, PLCG1, CD247 | 8.2 | 4.55E-06 |
| hsa04650 | Natural killer cell mediated cytotoxicity | 5.1 | KIR2DS4, SH2D1A, SH2D1B, PRF1, NFATC2, GZMB, KIR3DL1, FASLG, ZAP70, LCK, KLRD1, KLRC1, PLCG1, CD247 | 6.7 | 8.25E-06 |
| hsa04659 | Th17 cell differentiation | 4.7 | RORC, NFATC2, RORA, CD3G, GATA3, CD3E, CD3D, ZAP70, LCK, TBX21, IL2RB, PLCG1, CD247 | 7.0 | 1.44E-05 |
| hsa04640 | Hematopoietic cell lineage | 4.3 | CD2, ITGA4, CD8B, CD5, CD8A, CD7, IL5RA, CD3G, CD3E, IL7R, CD3D, MS4A1 | 7.4 | 2.13E-05 |
| hsa05235 | PD-L1 expression and PD-1 checkpoint pathway in cancer | 3.6 | ZAP70, LCK, CD28, NFATC2, CD3G, PLCG1, CD247, CD3E, CD3D, RASGRP1 | 6.4 | 8.10E-04 |
| hsa05340 | Primary immunodeficiency | 2.5 | ZAP70, CD8B, LCK, CD8A, CD3E, IL7R, CD3D | 10.8 | 0.0012 |
| hsa05332 | Graft-versus-host disease | 2.5 | CD28, PRF1, GZMB, KLRD1, KIR3DL1, FASLG, KLRC1 | 10.5 | 0.0012 |
| hsa05142 | Chagas disease | 3.3 | C1QA, ACE, PPP2R2B, FASLG, CD3G, CD247, CD3E, CD3D, C1QC | 5.1 | 0.0087 |
| hsa04514 | Cell adhesion molecules | 3.6 | CD2, SPN, CD6, ITGA4, CD8B, CD8A, CD28, NCAM1, ITGB7, TIGIT | 3.7 | 0.0261 |
| hsa04612 | Antigen processing and presentation | 2.5 | HSPA8, CD8B, CD8A, KIR2DS4, KLRD1, KIR3DL1, KLRC1 | 5.7 | 0.0261 |
| hsa05162 | Measles | 3.3 | HSPA8, CCND2, IL2RB, BCL2, CD28, FASLG, CD3G, CD3E, CD3D | 3.7 | 0.0500 |

Results from gene enrichment analysis with Kyoto Encyclopedia of Genes and Genomes (KEGG). All differentially expressed genes with a cut off FDR < 0.02 and positive fold change were included in the analysis. Pathway is the enriched KEGG pathway, sorted by significance. % is the percentage of differentially expressed genes belonging to the pathway. Genes are the gene symbol of the differentially expressed genes belonging to the pathway. Fold enrichment is the percentage of differentially expressed genes belonging to the pathway, divided by the corresponding percentage in the background. FDR is the significance level after correction for multiple testing with false discovery rate.

Table A3 Enriched GO biological processes from downregulated genes

| Term | Name | % | Genes | Fold Enrichment | FDR |
| --- | --- | --- | --- | --- | --- |
| GO:0050729 | positive regulation of inflammatory response | 4.5 | NFKBIA, IL1RL1, NLRP12, IL1B, OSM, NLRP3, TLR4, NAIP, TLR2, SNCA, LILRA5 | 8.0 | 0.0019 |
| GO:0006955 | immune response | 7.4 | IL10, IL1RN, IL1R1, IL1R2, OSM, IL1RAP, FCAR, TNFSF13B, IL1RL1, CLEC4D, IL18RAP, ITGAD, IL1B, FCGR1A, CLEC4E, TLR4, IL18R1, TLR2 | 3.9 | 0.0027 |
| GO:0006915 | apoptotic process | 9.8 | KLLN, PLK3, HIP1, BCL2A1, GADD45A, TNFAIP3, NR2E1, BMX, MAPK14, STK3, TNFRSF10D, NFKBIA, RNF144B, PLSCR1, MARCKS, DRAM1, CASP5, IL1B, DDIT4, NLRP3, KIF1B, NAIP, MAP2K6, TLR2 | 3.0 | 0.0027 |
| GO:0042742 | defense response to bacterium | 4.1 | IL10, PLAC8, CLEC4D, ANXA3, HP, FPR2, FCGR1A, CLEC4E, TLR4, NAIP | 6.2 | 0.0151 |
| GO:0051092 | positive regulation of NF-kappaB transcription factor activity | 4.5 | IL18RAP, IL1B, NLRP3, TRIM25, FLOT2, IRAK3, IL1RAP, TLR4, IL18R1, ICAM1, TLR2 | 5.2 | 0.0175 |
| GO:2000556 | positive regulation of T-helper 1 cell cytokine production | 1.6 | IL1R1, IL1B, ARID5A, IL18R1 | 37.5 | 0.0342 |

Results from gene enrichment analysis with Gene ontology (GO). All differentially expressed genes with a cut off FDR < 0.02 and negative fold change were included in the analysis, and all differentially expressed genes were used as background. Term and name are the enriched GO enriched biologic process using the DAVID default function BP_DIRECT, sorted by significance. % is the percentage of differentially expressed genes belonging to the pathway. Genes are the gene symbol of the differentially expressed genes belonging to the pathway. Fold enrichment is the percentage of differentially expressed genes belonging to the pathway, divided by the corresponding percentage in the background. FDR is the significance level after correction for multiple testing with false discovery rate.

Table A4 Enriched KEGG pathways from downregulated genes

| Pathway | Name | % | Genes | Fold Enrichment | FDR |
| --- | --- | --- | --- | --- | --- |
| hsa04064 | NF-kappa B signaling pathway | 4.5 | NFKBIA, BCL2A1, IL1R1, GADD45A, IL1B, TNFAIP3, TRIM25, CXCL1, TLR4, TNFSF13B, ICAM1 | 6.3 | 0.0017 |
| hsa05144 | Malaria | 2.9 | IL10, CR1, IL1B, HGF, TLR4, ICAM1, TLR2 | 8.2 | 0.0157 |
| hsa05140 | Leishmaniasis | 3.3 | IL10, NFKBIA, CR1, IL1B, FCGR1A, MAPK14, TLR4, TLR2 | 6.4 | 0.0157 |
| hsa05134 | Legionellosis | 2.9 | NFKBIA, CR1, IL1B, CXCL1, TLR4, NAIP, TLR2 | 7.2 | 0.0202 |
| hsa04668 | TNF signaling pathway | 3.7 | NFKBIA, MLKL, IL1B, TNFAIP3, CXCL1, MAPK14, IL18R1, ICAM1, MAP2K6 | 4.7 | 0.0255 |
| hsa04621 | NOD-like receptor signaling pathway | 4.5 | NFKBIA, NLRP12, CASP5, IL1B, NLRP3, TNFAIP3, CXCL1, MAPK14, TLR4, NAIP, IFNAR1 | 3.6 | 0.0279 |
| hsa04060 | Cytokine-cytokine receptor interaction | 5.7 | IL10, IL1RN, IL1R1, IL1R2, OSM, CXCL1, IL1RAP, TNFSF13B, TNFRSF10D, IL1RL1, IL18RAP, IL1B, IL18R1, IFNAR1 | 2.9 | 0.0279 |
| hsa04010 | MAPK signaling pathway | 5.7 | IL1R1, GADD45A, DUSP1, HGF, IL1RAP, MAPK14, CACNA1E, STK3, IL1B, MKNK1, PDGFC, RASA2, MAP3K20, MAP2K6 | 2.8 | 0.0369 |
| hsa05146 | Amoebiasis | 3.3 | IL10, LAMB3, IL1R1, IL1B, IL1R2, CXCL1, TLR4, TLR2 | 4.6 | 0.0373 |
| hsa04625 | C-type lectin receptor signaling pathway | 3.3 | IL10, NFKBIA, PLK3, CLEC4D, IL1B, NLRP3, MAPK14, CLEC4E | 4.5 | 0.0397 |

Results from gene enrichment analysis with Kyoto Encyclopedia of Genes and Genomes (KEGG). All differentially expressed genes with a cut off FDR < 0.02 and negative fold change were included in the analysis. Pathway is the enriched (KEGG) pathway, sorted by significance. % is the percentage of differentially expressed genes belonging to the pathway. Genes are the gene symbol of the differentially expressed genes belonging to the pathway. Fold enrichment is the percentage of differentially expressed genes belonging to the pathway, divided by the corresponding percentage in the background. FDR is the significance level after correction for multiple testing with false discovery rate.

Table A5 Differentially expressed pro -and anti-inflammatory single markers

| **Cytokines** | **log_2_ Fold change** | **FDR** |
| --- | --- | --- |
| IL32 | 0.262691 | 7.34E-08 |
| IL10 | -0.36224 | 6.76E-07 |
| TNFSF13B | -0.20329 | 2.45E-05 |
| IL1B | -0.15029 | 0.0078 |
| **Chemokines** |  |  |
| CXCL1 | -0.16357 | 0.0099 |
| PDGFC | -0.18925 | 0.0002 |
| FASLG | 0.31906 | 0.0182 |
| CD40LG | 0.18896 | 0.0495 |
| **Receptors** |  |  |
| IL1RAP | -0.34068 | 4.61E-15 |
| IL1R2 | -0.33862 | 4.76E-14 |
| IL2RB | 0.316337 | 5.03E-11 |
| TGFBR3 | 0.35722 | 3.72E-09 |
| IL18RAP | -0.25123 | 4.34E-08 |
| IL18R1 | -0.25027 | 7.36E-08 |
| IL5RA | 0.374828 | 2.03E-06 |
| IL7R | 0.181919 | 0.0004 |
| IL1RL1 | -0.2089 | 0.0007 |
| IL1R1 | -0.16969 | 0.0011 |
| TNFRSF10D | -0.21211 | 0.0016 |
| FGFR1 | 0.197644 | 0.0059 |
| IFNAR1 | -0.14498 | 0.0101 |
| TNFRSF25 | 0.196381 | 0.0117 |
| TLR4 | -0.14354 | 0.0133 |
| TNFRSF4 | 0.308128 | 0.0134 |
| TLR2 | -0.14119 | 0.0154 |
| IL23R | 0.941818 | 0.0201 |
| CXCR3 | 0.218487 | 0.0224 |
| CKCR4 | 0.131324 | 0.0290 |
| IL4R | -0.13249 | 0.0298 |
| TNFRSF18 | 0.355081 | 0.0321 |
| TLR1 | -0.12806 | 0.0378 |
| IL21R | 0.126321 | 0.1051 |
| **Receptor antagonist** |  |  |
| IL1RN | -0.15032 | 0.0074 |
| **Enzymes** |  |  |
| TNFAIP3 | -0.19353 | 9.69E-05 |
| **Cell adhesion molecules** |  |  |
| ICAM1 | -0.14876 | 0.0078 |
| NCAM1 | 0.239705 | 0.0097 |
